# Smoking, Green Tea Consumption, Genetic Polymorphisms in the Insulin-Like Growth Factors and Lung Cancer Risk

**DOI:** 10.1371/journal.pone.0030951

**Published:** 2012-02-07

**Authors:** I-Hsin Lin, Ming-Lin Ho, Hsuan-Yu Chen, Hong-Shen Lee, Chia-Chen Huang, Yin-Hung Chu, Shiau-Yun Lin, Ya-Ru Deng, Yu-Hao He, Yu-Hui Lien, Chi-Wen Hsu, Ruey-Hong Wong

**Affiliations:** 1 Institute of Public Health, Chung Shan Medical University, Taichung, Taiwan; 2 Institute of Medicine, Chung Shan Medical University, Taichung, Taiwan; 3 Division of Pulmonary Medicine, Department of Internal Medicine, Changhua Christian Hospital, Changhua, Taiwan; 4 Institute of Statistical Science, Academia Sinica, Taipei, Taiwan; 5 Department of Public Health, Chung Shan Medical University, Taichung, Taiwan; 6 Department of Occupational Medicine, Chung Shan Medical University Hospital, Taichung, Taiwan; Brigham and Women's Hospital and Harvard Medical School, United States of America

## Abstract

Insulin-like growth factors (IGFs) are mediators of growth hormones; they have an influence on cell proliferation and differentiation. In addition, IGF-binding protein (IGFBP)-3 could suppress the mitogenic action of IGFs. Interestingly, tea polyphenols could substantially reduce IGF1 and increase IGFBP3. In this study, we evaluated the effects of smoking, green tea consumption, as well as *IGF1*, *IGF2*, and *IGFBP3* polymorphisms, on lung cancer risk. Questionnaires were administered to obtain the subjects' characteristics, including smoking habits and green tea consumption from 170 primary lung cancer cases and 340 healthy controls. Genotypes for *IGF1*, *IGF2*, and *IGFBP3* were identified by polymerase chain reaction. Lung cancer cases had a higher proportion of smoking, green tea consumption of less than one cup per day, exposure to cooking fumes, and family history of lung cancer than controls. After adjusting the confounding effect, an elevated risk was observed in smokers who never drank green tea, as compared to smokers who drank green tea more than one cup per day (odds ratio (OR) = 13.16, 95% confidence interval (CI) = 2.96–58.51). Interaction between smoking and green tea consumption on lung cancer risk was also observed. Among green tea drinkers who drank more than one cup per day, *IGF1* (CA)_19_/(CA)_19_ and (CA)_19_/X genotypes carriers had a significantly reduced risk of lung cancer (OR = 0.06, 95% CI = 0.01–0.44) compared with *IGF1* X/X carriers. Smoking-induced pulmonary carcinogenesis could be modulated by green tea consumption and their growth factor environment.

## Introduction

Lung cancer is the most frequently occurring cancer worldwide [1], and it has a well established correlation with cigarette smoking [2]. Free radicals have been also implicated in smoking-related carcinogenesis [3], [4]. Thus, an oxidant/antioxidant imbalance may play a role in lung carcinogenesis among those exposed to cigarette smoke.

Tea is consumed largely in Asian countries, such as Japan, China and Taiwan [5]. Tea has received a great deal of attention because tea polyphenols are strong antioxidants. Experimental studies have shown consistently that green tea may inhibit the induction of a number of cancers [6], [7]. However, the mechanisms of these inhibitory effects are not clear, and epidemiological studies of tea consumption and cancer are limited and results are inconclusive [8]–[10].

Insulin-like growth factors (IGFs), IGF1 and IGF2 are peptide hormones with strong mitogenic effect on both normal and cancerous cells, including lung cancer [11], [12]. Their major binding protein, IGF-binding protein 3 (IGFBP3), not only regulates the mitogenic action of IGFs but also inhibits their antiapoptotic effect [11]. Besides its IGF-dependent function, IGFBP3 also has an IGF independent inhibitory effect on cell growth [11]. The level of IGFBP3 in serum, in some situations, suppresses the mitogenic action of IGFs, and is inversely associated with cancer risk [13]. Previous studies of the relationship of smoking and IGFs levels, however, have yielded inconsistent results [14], [15]. In addition, tea polyphenols are associated with a substantial reduction in IGF1 levels and an increase in IGFBP3 levels in TRAMP mice [16] as well as human colon cell lines [17]. Therefore, tea polyphenols may decrease lung cancer risk by increasing IGFBP3 levels and lowering IGFs.

Rosen et al. [18] first reported that the (CA)_19_/(CA)_19_ genotype for the *IGF1* microsatellite polymorphism (cytosine-adenosine, or CA dinucleotide repeat) was associated with decreased levels of serum IGF1. In addition, a cohort study of over 2,500 Caucasian males established a relationship between an *Apa*I polymorphism (rs680) in the *IGF2* gene region of chromosome 11p15 and serum IGF2 concentrations [19]. Deal et al. [20] also indicated that several single-nucleotide polymorphisms in the promoter of *IGFBP3*, and documented *in vitro* significantly higher promoter activity of the A allele at the -202 locus (rs2854744) compared with the C allele, consistent with the relationship observed between genotype and circulating IGFBP3.

Here, we evaluated the effects of smoking, green tea consumption, and *IGF1*, *IGF2*, and *IGFBP3* genotypes on lung cancer risk. We also assessed the joint effects of smoking and green tea consumption, and smoking and *IGFs* and *IGFBP3* genotypes on lung cancer risk. Then, we examined the effect of *IGFs* and *IGFBP3* on lung cancer stratified by habits of green tea consumption.

## Materials and Methods

### Study design

Our entire study in which experiments on patients or healthy volunteers, patient's case histories conformed to the Declaration of Helsinki, and their consent statement was written. The design of the work and final report were approved by the institutional review board of participating institutions.

From August 2004 to October 2008, a total of 241 lung cancer patients (International Classification of Diseases, 9th revision; ICD code 162) were recruited from Changhwa Christian Hospital (Changhwa County, Taiwan). The hospital was accessible to patients from all socioeconomic classes. All patients underwent a series of examinations of pathologic stages by board-certified pathologists. Tumor types and stages were determined according to the World Health Organization classification [21]. Ten patients were not interviewed because of severe illness, 37 were not interviewed because they were not the incident cases, and 24 were excluded due to older age (range = 80–92 years) or incomplete or missing questionnaire data. Among 170 patients available for matching, cell types included 93 adenocarcinoma (54.7%), 46 squamous cell carcinoma (27.1%), and 31 others (including small cell carcinoma, neuroendocrine carcinoma, mixed cell carcinoma, and unspecific malignant cell). For this case-control study, two controls were selected for each lung cancer case, matched by gender and age (±5 years). Therefore, 340 controls were randomly selected from consecutive patients with no history of cancer who were admitted to the same teaching medical center for physical check-up.

### Epidemiological information

Epidemiological information was collected from study subjects by in-person interviews using a standardized questionnaire. The questionnaire contained questions involving demographic characteristic and lifestyle. Subjects' smoking habits included the number of cigarettes smoked daily and the length of time smoking. The parameter “pack-years” were used to indicate cumulative smoking dose, defined as the number of packs of cigarettes smoked daily multiplied by the number of years of active smoking. In Taiwan, tea is the most commonly made in a small earthenware teapot. Tea is served in small cups (30–50 ml), and the same tea leaf can be brewed many times. In this study, we defined a standardized cup of tea as 100–120 ml. The period of exposure was assessed from birth to the day when lung cancer was first diagnosed (for cases) or when the interview was performed (for controls). To prevent misclassification of tea intake for individuals who changed their tea consumption after diagnosis, we collected only cumulative amounts before disease onset. Subjects who reported drinking green tea were further asked the length (number of years) and frequency of green tea consumption: every day (≥1 cup per day), 3–4 cups per week, 1–2 cups per week, 1–2 cups per month and seldom. Those who consumed tea every day were asked the number of cups drunk: <1, 1–2, 3–4, 5–9, or 10+ per day, based on a previous study [10]. Intake of fruits and vegetables was measured as the average number of standardized servings per week of fruits and vegetables over the last 3 years. For cooking exposures, subjects were asked the frequency of using various cooking methods, such as stir-frying. Family history of lung cancer was defined as lung cancer in first-degree relatives of the test subject.

### Genotyping analysis of *IGF1, IGF2,* and *IGFBP3*

Venous blood was collected into heparinized tubes from all subjects, and was prepared into plasma, buffy coat and red blood cells. Genomic DNA was extracted from buffy coat. Genotype was analyzed using polymerase chain reaction (PCR)-based methods as described below. The *IGF1* (CA)_n_ repeat was determined by PCR amplification of the polymorphic region as described by Rosen et al. [18]. A microsatellite located in the promoter of the *IGF1* gene was used to type the *IGF1* gene. Primers used to amplify the *IGF1* gene were 5′-GCT AGC CAG CTG GTG TTA TT-3′ and 5′-ACC ACT CTG GGA GAA GGG TA-3; the forward primer was labeled with a fluorescent dye (FAM). One half microliter of DNA was added to a PCR buffer containing 200 ng of primers, 1.5 mM MgCl_2_, 0.2 mM of dNTPs, 50 mM KCl, 10 mM Tris-HCl (pH 8.3), and 0.1% of bovine serum albumin in a final volume of 50 µl. The cycling parameters consisted of an initial incubation of 5 min at 94°C, followed by 35 cycles of 1 min at 94°C (denaturing step), 1 min at 57°C (annealing), and 1 min at 72°C (extension). The lengths of fragments were assigned by short tandem repeat polymorphism using the ABI 3730 DNA sequencer and Genemapper Software (Applied Biosystems, Foster City, CA, USA). This genotyping was done by the National Genotyping Center, Institute of Biomedical Sciences, Academia Sinica (Taipei, Taiwan). Furthermore, representative homozygotes were sequenced to confirm (CA)_n_ repeat numbers from base pair lengths, using ABI 3730 DNA sequencer and Chromas Lite 2.0 for Windows Software (Technelysium Pty Ltd, Eden Prairie, MN, USA).

For *IGF2* gene analysis, restriction fragment length polymorphism (RFLP) was detected by differences in *Apa*I at 820 locus (rs680) of *IGF2* following PCR amplification [22]. Primers used to amplify the *IGF2* gene were 5′-GCA CAG CAG CAT CTT CAA ACA TG-3′ and 5′-GGG TCG TGC CAA TTA CAT TTC AT-3′. The cycling parameters consisted of an initial incubation of 5 min at 94°C, followed by 35 cycles of 30 s at 94°C, 30 s at 65°C, and 1 min at 72°C. The PCR products were digested with *Apa*I. Homozygous AA individuals exhibited a product fragment of 620 bp, homozygous GG individuals had a 390 bp and 230 bp fragment, and heterozygous AG individuals had all three fragments. The *IGFBP3 Fsp*I polymorphism (rs2854744) was determined by the methods developed by Ren et al. [23]. Primers used to amplify the *IGFBP3* gene were 5′-GAA TGC GGA GCG CTG TAT G-3′ and 5′-TGT GGA ATC CAG GCA GGA AG-3′. The cycling parameters consisted of an initial incubation of 4 min at 94°C, followed by 35 cycles of 1 min at 94°C, 1 min at 65°C, and 1 min at 72°C. The PCR products were digested with *Fsp*I. Homozygous AA individuals exhibited a product fragment of 483 bp, homozygous CC individuals had a 258 bp and 225 bp fragment, and heterozygous AC individuals had all three fragments.

### Statistical analysis

Comparisons between case and control groups for gender, age, smoking status, pack-years of smoking, green tea consumption, dietary intake of fruits and vegetables, exposure to cooking fumes, and family history of lung cancer were made using Student's *t*-test for continuous variables and χ^2^ test or Fisher's exact test for discrete variables. χ^2^ test was also used to test the prevalence of *IGF1* (CA)_n_ repeat, *IGF2* 820 and *IGFBP3* -202 genotypes between case and control groups. A conditional logistic regression model was employed to obtain the adjusted odds ratio (OR) and 95% confidence interval (CI) for each variable. Likelihood ratio χ^2^ tests were also utilized to test the interaction between smoking and green tea consumption, and the interaction between smoking and *IGFs* and *IGFBP3* genotypes with respect to the risk of lung cancer. Associations between lung cancer and *IGFs* and *IGFBP3* genotypes stratified by amount of green tea consumed were also assessed. A P value <0.05 was considered to indicate statistical significance, and all tests were two-tailed. The type Ιerror rate were adjusted by multiple testing (bonferroni correction). All data were analyzed using SAS 9.1 (SAS, Inc., Cary, NC, USA).

## Results

The specific characteristics of study subjects were summarized in Table 1. In total, 510 study subjects (306 males and 204 females) participated in this study. Mean age was 66.2 years for lung cancer patients (range = 38–90 years) and 64.6 years for control subjects (range = 38–87 years) at recruitment. More smokers were represented in the lung cancer cases compared with the controls (54.1% versus 27.1%; P<0.01), 37.6% of smokers with lung cancer smoked more than 40 pack-years, but only 14.7% of controls who smoked. Besides, the groups were significantly different in green tea consumption. Compared with controls, more cases were green tea nondrinkers (85.3% versus 66.5%; P<0.01). Only a few cases (5.3%) consumed green tea more than ten years, compared to 16.5% of controls (P<0.01). In addition, more cases than controls were exposed to cooking fumes ≥1 hour per week (20.6% versus 8.2%; P<0.01).

**Table 1:** Frequency distribution of specific characteristics by case and control status.

Variable	Cases	Controls	OR (95% CI)	P-value^1^
	(n = 170)	(n = 340)		
**Gender**				
Male	102 (60.0%)	204 (60.0%)	1.00 (ref.)	1.00
Female	68 (40.0%)	136 (40.0%)	1.00 (0.69–1.46)	
**Age at recruitment (years ± SD)**	66.2±10.6	64.6±10.6		0.12
≤50	16 (9.4%)	34 (10.0%)	1.00 (ref.)	0.59
51–59	29 (17.1%)	70 (20.6%)	0.88 (0.42–1.84)	
≥60	125 (73.5%)	236 (69.4%)	1.13 (0.60–2.12)	
**Smoking status**				
Never smokers	78 (45.9%)	248 (72.9%)	1.00 (ref.)	<0.01
Current and ever smokers	92 (54.1%)	92 (27.1%)	7.61 (3.98–14.56)	
**Pack-years smoked**				
0	78 (45.9%)	248 (72.9%)	1.00 (ref.)	<0.01
1–39	28 (16.5%)	42 (12.4%)	4.79 (2.25–10.21)	
≥40	64 (37.6%)	50 (14.7%)	10.37 (5.07–21.24)	
**Green tea consumption (cups/day)**				
≥1	7 (4.1%)	64 (18.8%)	1.00 (ref.)	<0.01
<1	18 (10.6%)	50 (14.7%)	3.01 (1.13–8.05)	
0	145 (85.3%)	226 (66.5%)	6.34 (2.69–14.91)	
**Green tea consumption (years)**				
>10	9 (5.3%)	56 (16.5%)	1.00 (ref.)	<0.01
≤10	16 (9.4%)	58 (17.1%)	1.90 (0.77–4.70)	
0	145 (85.3%)	226 (66.5%)	4.96 (2.22–11.04)	
**Vegetables and fruits intakes (servings/week)**				
≥21	86 (50.6%)	175 (51.5%)	1.00 (ref.)	0.36
15–20	42 (24.7%)	67 (19.7%)	1.28 (0.55–1.37)	
≤14	42 (24.7%)	98 (28.8%)	0.87 (0.79–2.06)	
**Exposure to fume of cooking (hours/week)**				
<1	135 (79.4%)	312 (91.8%)	1.00 (ref.)	<0.01
1–3	17 (10.0%)	16 (4.7%)	2.89 (1.33–6.29)	
≥3	18 (10.6%)	12 (3.5%)	3.80 (1.72–8.38)	
**Family history of lung cancer**				
No	160 (94.1%)	334 (98.2%)	1.00 (ref.)	0.01
Yes	10 (5.9%)	6 (1.8%)	3.74 (1.27–11.02)	

Data were matched by age and gender, calculated by conditional logistic regression.

1 Two-sided χ^2^ test or Fisher's exact test for discrete variables and paired *t*-test for continuous variables.

Table 2 shows the genotypic prevalence of *IGF1* (CA)_n_ repeat, *IGF2* 820, *IGFBP3* -202 among study subjects. Among subjects, the number of repeats for the *IGF1* (CA)_n_ polymorphism ranged 13–25, and the most common allele was (CA)_19_ among both cases and controls. The frequency for *IGF1* (CA)_19_/(CA)_19_ and (CA)_19_/X genotypes in cases was significantly lower than in controls (51.8% versus 62.9%; P = 0.02). The prevalence of *IGF2* and *IGFBP3* genotypes in case and control groups was not significantly different. Frequencies for the *IGF1*, *IGF2*, and *IGFBP3* genotypes were in Hardy-Weinberg equilibrium in the control group.

**Table 2:** Genotype frequencies of *IGF1* (CA)_n_ repeat, *IGF2* 820 and *IGFBP3* -202 among cases and controls.

Genotype	Cases	Controls	OR (95% CI)	P-value^1^	P-value^2^
	(n = 170)	(n = 340)			
***IGF1*** ** (CA)_n_ repeat (%)**					
(CA)_19_/(CA)_19_	23 (13.5%)	43 (12.6%)	1.00 (ref.)	0.03	0.20
(CA)_19_/X	65 (38.2%)	171 (50.3%)	0.71 (0.39–1.27)		
X/X	82 (48.3%)	126 (37.1%)	1.23 (0.69–2.20)		
(CA)_19_/(CA)_19_+(CA)_19_/X	88 (51.8%)	214 (62.9%)	1.00 (ref.)	0.02	
X/X	82 (48.2%)	126 (37.1%)	1.61 (1.10–2.34)		
(CA)_19_ allele	111 (32.6%)	257 (37.8%)	1.00 (ref.)	0.11	
X allele	229 (67.4%)	423 (62.2%)	1.26 (0.96–1.66)		
***IGF2*** ** 820 (%)**					
GG	35 (20.6%)	85 (25.0%)	1.00 (ref.)	0.42	0.04
AG	75 (44.1%)	151 (44.4%)	1.17 (0.73–1.90)		
AA	60 (35.3%)	104 (30.6%)	1.37 (0.83–2.25)		
GG	35 (20.6%)	85 (25.0%)	1.00 (ref.)	0.27	
AA+AG	135 (79.4%)	255 (75.0%)	1.26 (0.82–1.95)		
G allele	145 (42.6%)	321 (47.2%)	1.00 (ref.)	0.17	
A allele	195 (57.4%)	359 (52.8%)	0.84 (0.64–1.09)		
***IGFBP3*** ** -202 (%)**					
CC	14 (8.2%)	36 (10.6%)	1.00 (ref.)	0.62	0.28
AC	66 (38.8%)	136 (40.0%)	1.25 (0.63–2.47)		
AA	90 (53.0%)	168 (49.4%)	1.38 (0.71–2.70)		
CC	14 (8.2%)	36 (10.6%)	1.00 (ref.)	0.40	
AA+AC	156 (91.8%)	304 (89.4%)	1.32 (0.69–2.52)		
C allele	94 (27.6%)	208 (30.6%)	1.00 (ref.)	0.33	
A allele	246 (72.4%)	472 (69.4%)	1.16 (0.87–1.55)		

Data were matched by age and gender, calculated by conditional logistic regression.

1 Data were calculated by two-sided χ^2^ test.

2 The probability of the χ^2^ test for Hardy-Weinberg equilibrium in control group.

Further, we analyzed the association between green tea consumption and lung cancer risk (Table 3). Subjects were stratified into nonsmokers and smokers. The results showed a clear association between green tea consumption and lung cancer risk in current and ever smokers who drank less green tea (<1 cup per day and never drank green tea, P for trend <0.0001). After adjusting for exposure to cooking fumes and family history of lung cancer, we defined the smokers who drank ≥1 cup per day of green tea as the reference group (OR = 1.00). Compared with the reference group, smokers who drank <1 cup per day of green tea had an OR of 13.32 (95% CI = 2.79–63.61) for lung cancer; smokers who never drank green tea had a 13.16-fold (95% CI = 2.96–58.51) greater risk of lung cancer. Smokers who never drank green tea compared to smokers who drank green tea for >10 years also had a significantly greater risk of lung cancer (OR = 3.34, 95% CI = 1.41–7.93). A significant interaction between smoking status and green tea consumption (number of cups per day) on lung cancer risk was also observed.

**Table 3:** Association between green tea consumption and smoking status with lung cancer risk.

Variable	Smoking status					
	Never smokers			Current and ever smokers		
	CA	CN	OR (95% CI)	CA	CN	OR (95% CI)
**Green tea consumption (cups/day)**						
≥1	4	31	1.00 (ref.)	3	33	1.00 (ref.)
<1	3	38	0.69 (0.14–3.39)	15	12	13.32 (2.79–63.61)^1^
0	71	179	1.95 (0.65–5.85)	74	47	13.16 (2.96–58.51)^1^
P for trend			0.0025			<0.0001
Test for interaction	χ^2^ = 7.24 (2 df); P = 0.03					
**Green tea consumption (years)**						
>10	2	29	1.00 (ref.)	7	27	1.00 (ref.)
≤10	5	40	1.80 (0.33–9.75)	11	18	2.19 (0.76–6.28)
0	71	179	3.22 (0.76–13.58)	74	47	3.34 (1.41–7.93)^2^
P for trend			0.0025			<0.0001
Test for interaction	χ^2^ = 0.13 (2 df); P = 0.94					

Data were matched by age and gender, calculated by multiple conditional logistic regression and adjusted for exposure to fume of cooking, and family history of lung cancer.

1 P<0.01,

2 P = 0.04; P value was adjusted by multiple testing (bonferroni correction).

Subsequently, we analyzed the joint effect of cumulative smoking dose with *IGF1*, *IGF2*, and *IGFBP3* genotypes on lung cancer risk after adjusting for the effect of green tea consumption, exposure to cooking fumes, and family history of lung cancer (Table 4). Smoking status was stratified into 0, 1–39, ≥40 pack-years smoked. We selected nonsmokers carrying the *IGF1* (CA)_19_/(CA)_19_ and (CA)_19_/X genotypes as the reference group (OR = 1.00). Nonsmokers carrying the *IGF1* X/X genotype had an OR of 1.29 (95% CI = 0.78–2.15) for lung cancer. Among those who smoked for 1–39 pack-years, those carrying the *IGF1* (CA)_19_/(CA)_19_ and (CA)_19_/X genotypes (OR = 3.78, 95% CI = 1.79–8.01) and *IGF1* X/X genotype (OR = 4.51, 95% CI = 1.99–10.22) had significantly increased risks of lung cancer. Among those who smoked ≥40 pack-years, those carrying *IGF1* (CA)_19_/(CA)_19_ and (CA)_19_/X genotypes (OR = 6.14, 95% CI = 3.10–12.15) and *IGF1* X/X genotype (OR = 5.43, 95% CI = 2.84–10.36) also had significantly increased risk of lung cancer. However, the test for interaction between cumulative smoking dose and *IGF1* genotypes on lung cancer risk was not significant. Similar results also showed a significant combined effect of cumulative smoking dose and *IGF2* and *IGFBP3* genotypes.

**Table 4:** The joint effect of cumulative smoking dose with *IGF1* (CA)_n_ repeat, *IGF2* 820, *IGFBP3* -202 genotypes for lung cancer risk.

Variable	Pack-years smoked								
	0			1–39			≥40		
	CA	CN	OR (95% CI)	CA	CN	OR (95% CI)	CA	CN	OR (95% CI)
***IGF1*** ** (CA)_n_ repeat genotypes**									
(CA)_19_/(CA)_19_+(CA)_19_/X	42	163	1.00 (ref.)	16	29	3.78 (1.79–8.01)^1^	30	22	6.14 (3.10–12.15)^1^
X/X	36	85	1.29 (0.78–2.15)	12	13	4.51 (1.99–10.22)^1^	34	28	5.43 (2.84–10.36)^1^
Test for interaction	χ^2^ = 1.05 (2 df); P = 0.59								
***IGF2*** ** 820 genotypes**									
GG	18	61	1.00 (ref.)	6	11	4.09 (1.34–12.54)^2^	11	13	5.83 (2.28–14.93)^1^
AA+AG	60	187	1.09 (0.58–2.06)	22	31	3.89 (1.71–8.83)^1^	53	37	5.50 (2.55–11.89)^1^
Test for interaction	χ^2^ = 0.52 (2 df); P = 0.77								
***IGFBP3*** ** -202 genotypes**									
CC	6	27	1.00 (ref.)	1	4	2.05 (0.22–19.00)	7	5	4.71 (1.36–16.32)^2^
AA+AC	72	221	1.16 (0.46–2.90)	27	38	4.43 (1.59–12.34)^3^	57	45	6.13 (2.23–16.85)^1^
Test for interaction	χ^2^ = 0.79 (2 df); P = 0.67								

Data were matched by age and gender, calculated by multiple conditional logistic regression and adjusted for green tea consumption, exposure to fume of cooking, and family history of lung cancer.

1 P<0.01,

2 P = 0.08,

3 P = 0.03; P value was adjusted by multiple testing (bonferroni correction).

We also evaluated the effect of *IGF1* (Table 5), *IGF2*, and *IGFBP*3 (data not shown) on lung cancer risk by green tea consumption. After adjusting for the effect of pack-years smoked, exposure to cooking fumes, and family history of lung cancer, we selected the *IGF1* X/X genotype carriers as the reference group for all green tea drinkers. Among green tea drinkers who drank ≥1 cup per day, *IGF1* (CA)_19_/(CA)_19_ and (CA)_19_/X genotypes carriers had a significantly reduced risk of lung cancer (OR = 0.06, 95% CI = 0.01–0.44). Those with *IGF1* (CA)_19_/(CA)_19_ and (CA)_19_/X genotypes who drank green tea for >10 years had a significantly reduced lung cancer risk (OR = 0.34, 95% CI = 0.12–0.95). However, no significant decrease in risk of lung cancer was found for green tea drinkers with *IGF2* and *IGFBP3* genotypes (data not shown).

**Table 5:** Risk of lung cancer in association with *IGF1* (CA)_n_ repeat genotype by different status of green tea consumption.

Variable	*IGF1* X/X genotype			*IGF1* (CA)_19_/(CA)_19_+(CA)_19_/X genotypes		
	CA	CN	OR (95% CI)	CA	CN	OR (95% CI)
**Green tea consumption (cups/day)**						
0	68	86	1.00 (ref.)	77	140	1.34 (0.93–1.91)
<1	9	18	1.00 (ref.)	9	32	0.94 (0.44–2.02)
≥1	5	22	1.00 (ref.)	2	42	0.06 (0.01–0.44)^1^
**Green tea consumption (years)**						
0	68	86	1.00 (ref.)	77	140	1.34 (0.93–1.91)
≤10	11	16	1.00 (ref.)	5	42	0.34 (0.12–0.95)^2^
>10	3	24	1.00 (ref.)	6	32	0.44 (0.19–1.03)^2^

Data were matched by age and gender, calculated by multiple conditional logistic regression and adjusted for pack-years of smoking, exposure to fume of cooking, and family history of lung cancer.

1 P = 0.03,

2 P = 0.11; P value was adjusted by multiple testing (bonferroni correction).

## Discussion

Our study found a protective effect of green tea consumption on lung cancer elicited by cigarette smoking. Green tea drinkers with susceptible *IGF1* (CA)_19_/(CA)_19_ and (CA)_19_/X genotypes had a reduced risk of lung cancer.

Tea polyphenols may inhibit the induction of a variety of cancers, including lung cancer [6], [7]. Polyphenols appear to be strong antioxidants and may also prevent mutagenicity and genotoxicity, inhibit tumor initiation, promotion, and cell proliferation, modulate detoxification enzymes, and trap activated metabolites of carcinogens [6]. In different animal models, oolong tea, jasmine tea, and green tea all inhibited lung tumorigenesis induced by a variety of carcinogens, including nicotine-derived nitrosamine ketone [6]. Our results suggest that individuals who never drank green tea have an elevated lung cancer risk compared to those who drank green tea at least one cup per day, and the effect is more pronounced in smokers. However, epidemiological studies of tea consumption and cancer are limited and the results are inconclusive [8]–[10]. Although our finding of an inverse association of green tea consumption with the risk of lung cancer is consistent with previous experimental and epidemiological studies [9], [10], an alternative explanation for the observed association is uncontrolled confounding factor.

The *IGF1* (CA)_19_/(CA)_19_ genotype was reported previously to correlate with lower serum level of IGF1 [18]. The *IGF2* AA genotype has been suggested to correlate with higher serum level of IGF2 [19], and the *IGFBP3* C allele to correlate with lower plasma levels of IGFBP3 [20]. A previous study proposed that higher IGF1 serum levels and lower IGFBP3 levels were associated with cancer risk [15]; however, the intra-individual variability in circulating IGF1, IGF2, and IGFBP3 levels is genetically determined [18]–[20]. Thus, the association between *IGF1*, *IGF2*, and *IGFBP3* and cancer risk could be evaluated on the molecular level. In our study, the frequency for *IGF1* (CA)_19_/(CA)_19_ and (CA)_19_/X genotypes in cases (38.2%) was significantly lower than in controls (50.3%). Previous studies also have demonstrated a protective effect of *IGF1* (CA)_19_ allele on cancers, including breast, prostate and colorectal cancers [24]. However, we did not observe an independent relationship between the *IGF2* and *IGFBP3* polymorphisms and risk of lung cancer.

We investigated the joint effects of smoking and *IGF1*, *IGF2*, and *IGFBP3* genotypes on lung cancer, but no significant interaction between cumulative smoking dose and *IGF1*, *IGF2*, and *IGFBP3* genotypes on lung cancer was observed. Several studies have reported the association between smoking and IGF1 levels; however, results were inconsistent. A study by Kaklamani et al. [14] showed a positive association between IGF1 levels and pack-year history of smoking, but not with the number of cigarettes currently smoked. A case control study by Yu et al. [15] found no association between smoking status, pack-years of smoking, number of years smoking, total number of cigarettes currently smoked, and levels of IGF1 and IGFBP3 among control subjects. Although the relationship of smoking and IGF level is unclear, smoking may influence IGF physiology by influencing the circulating levels of IGF. Regardless, heavy smoking in cumulative dose would likely increase lung cancer risk by increasing carcinogen exposure long-term, while simultaneously increasing IGF1 or IGF2 levels or decreasing IGFBP3 levels. In keeping with this speculation, we observed that heavy smokers with susceptible *IGF1*, *IGF2*, and *IGFBP3* genotypes had an elevated risk of lung cancer, although the interaction effect was not significant (Table 4). The possibility of an association between smoking, IGF1 level and *IGF1* genotype on lung cancer risk needs further investigation.

We also evaluated the effect of *IGF1* on lung cancer risk by green tea consumption. Interestingly, we observed that green tea drinkers carrying *IGF1* (CA)_19_/(CA)_19_ and (CA)_19_/X genotypes had a significantly reduced risk of lung cancer compared with those carrying the *IGF1* X/X genotype. Individuals with *IGF1* (CA)_19_/(CA)_19_ and (CA)_19_/X genotypes who drank more than one cup of green tea per day also had significantly reduced lung cancer risk. Experimental studies have shown that tea polyphenols substantially reduce the levels of IGF1 in TRAMP mice [16] as well as human colon cell lines [17]. Thus, tea polyphenols may decrease lung cancer risk by lowering IGF1 levels [25]. Overall, our results suggest that the inverse association of lung cancer risk and green tea drinkers may be modulated by the IGFs system consistent with experimental findings. The protective effect of green tea consumption may be more evident in those with lower transcription activity of *IGF1*. However, our results should be elucidated by larger studies.

The present study revealed that the (CA)_19_ allele frequency for *IGF1* is 37.8% in the Taiwanese population, similar to that in other Asian populations (39.0%–40.8%) [26], [27], but significantly lower than in whites (64%–70.2%) [26], [27]. The A allele frequency for *IGF2* is 52.8% in Taiwan, similar to the 50% seen in Korea [28]; however, this is significantly higher than in Caucasian population (28.0%) [29]. The A allele frequency for *IGFBP3* is more common in the Taiwanese (69.4%) and Japanese populations (75.2%) [30] than in the American populations (whites 40.9% and blacks 53.1%) [31]. All genotype distributions in our healthy controls were in Hardy-Weinberg equilibrium. However, *IGF1*, *IGF2*, and *IGFBP3* may be susceptibility genes for lung cancer only in certain ethnic populations.

In the current study, recall bias may be a potential problem. Thus, misclassification of exposure may have influenced the effect of cigarette smoking on lung cancer risk. In addition, although the current study showed a protective effect of drinking green tea on lung cancer, several other reasons may explain the inconsistency between bioassays and the epidemiological data. First, quantification of green tea consumption in epidemiological studies, including this one, is subject to exposure misclassification that would tend to obscure any association. In our study, consumption of green tea was evaluated based on a previous study [10], in which spearman's coefficient for the correlation between the amounts of green tea consumed according to the questionnaire and the amounts consumed according to the three day in one year food records was 0.66, and the correlation between consumption measured by the two questionnaires administered six months apart was also 0.66. Since this may have resulted in non-differential misclassification, it is likely that the current findings are underestimated. Similar methods to assess green tea consumption have been employed in other studies [32], [33], implying the reasonable validity of this assessment. Neither cases nor controls were aware of study interest in green tea consumption. Thus, it is unlikely that participants provided biases information on green tea. Second, the concentrations of tea polyphenols used in the bioassays tend to be much higher than those to which humans are exposed in their routine consumption of green tea. Third, the bioavailability of polyphenols from green tea consumption may be very limited, since they are rapid metabolized and excreted. Several potential biomarkers of exposure to tea polyphenols, such as 4-*O*-methylgallic acid and isoferulic acid, have been proposed [34]. Unfortunately, no data was available on biomarkers for tea polyphenol exposure in our study. In addition, previous epidemiological studies may have obscured an association because the chemopreventive effects of green tea may be limited to genetically susceptible sub-populations. Our study was limited by the small numbers of subjects might generate a false positive result, especially in the analysis of subgroup. A larger study that includes a sufficient number of participants has the potential to show dose-effect relationships. Among 170 patients available for matching, the frequency of major cell types (adenocarcinoma, 54.7%; squamous cell carcinoma, 27.1%) was similar to that reported in a previous study conducted in Taiwan [35]. The male-to-female ratio of lung cancer in Taiwanese is approximately at 2∶1 [35]. In our present study, the proportion of males with lung cancer was 60%, which was close to general population in Taiwan. The mean age of our lung cancer patients at recruitment was 66.2 years was also comparable to that found in a previous study for Taiwanese (63.4 years) [35]. In addition, prevalence of cigarette smoking in our study subjects (cases, 54.1%; controls, 27.1%) was consistent with the findings of a previous study in Taiwanese (cases, 52.4%; controls, 29.9%) [36]. Therefore, our study subjects could be regarded to as representative of the general population of Taiwan. However, selection bias could have occurred when the healthy persons for physical check-up may have healthier behavior were enrolled as our study subjects.

Our study suggests an protective effect of green tea on lung cancer elicited by cigarette smoking. Green tea drinkers with susceptible *IGF1* genotypes have a reduced risk of lung cancer. Heavy smokers carrying susceptible *IGF1*, *IGF2*, and *IGFBP3* have a higher risk of lung cancer. This result may indicate that smoking-induced pulmonary carcinogenesis may be modulated by green tea consumption and the growth factor environment.

## References

[1] Ferlay J, Shin HR, Bray F, Forman D, Mathers C (2010). Estimates of worldwide burden of cancer in 2008: GLOBOCAN 2008.. Int J Cancer.

[2] Thun MJ, Henley SJ, Calle EE (2002). Tobacco use and cancer: an epidemiologic perspective for geneticists.. Oncogene.

[3] Church DF, Pryor WA (1985). Free-radical chemistry of cigarette smoke and its toxicological implications.. Environ Health Perspect.

[4] Pryor WA (1987). Cigarette smoke and the involvement of free radical reactions in chemical carcinogenesis.. Br J Cancer Supp.

[5] Graham HN (1992). Green tea composition, consumption, and polyphenol chemistry.. Prev Med.

[6] Yang CS, Wang ZY (1993). Tea and cancer.. J Natl Cancer Inst.

[7] Shankar S, Ganapathy S, Srivastava RK (2007). Green tea polyphenols: biology and therapeutic implications in cancer.. Front Biosci.

[8] Li Q, Kakizaki M, Kuriyama S, Sone T, Yan H (2008). Green tea consumption and lung cancer risk: the Ohsaki study.. Br J Cancer.

[9] Kinlen LJ, Willows AN, Goldblatt P, Yudkin J (1988). Tea consumption and cancer.. Br J Cancer.

[10] Tsubono Y, Nishino Y, Komatsu S, Hsieh CC, Kanemura S (2001). Green tea and the risk of gastric cancer in Japan.. New Engl J Med.

[11] Yu H, Rohan T (2000). Role of the insulin-like growth factor family in cancer development and progression.. J Natl Cancer Inst.

[12] Macauly VM (1992). Insulin-like growth factors and cancer.. Br J Cancer.

[13] Hernandez W, Grenade C, Santos ER, Bonilla C, Ahaghotu C (2007). IGF-1 and IGFBP-3 gene variants influence on serum levels and prostate cancer risk in African-Americans.. Carcinogenesis.

[14] Kaklamani VG, Linos A, Kaklamani E, Markaki I, Mantzoros C (1999). Age, sex, and smoking are predictors of circulating insulin-like growth factor 1 and insulin-like growth factor-binding protein 3.. J Clin Oncol.

[15] Yu H, Spitz MR, Mistry J, Gu J, Hong WK (1999). Plasma levels of insulin-like growth factor-I and lung cancer risk: a case-control analysis.. J Natl Cancer Inst.

[16] Adhami VM, Siddiqui IA, Ahmad N, Gupta S, Mukhtar H (2004). Oral consumption of green tea polyphenols inhibits insulin-like growth factor-I-induced signaling in an autochthonous mouse model of prostate cancer.. Cancer Res.

[17] Shimizu M, Deguchi A, Hara Y, Moriwaki H, Weinstein IB (2005). EGCG inhibits activation of the insulin-like growth factor-1 receptor in human colon cancer cells.. Biochem Biophys Res Commun.

[18] Rosen CJ, Kurland ES, Vereault D, Adler RA, Rackoff PJ (1998). Association between serum insulin growth factor-I (IGF-I) and a simple sequence repeat in IGF-I gene: implications for genetic studies of bone mineral density.. J Clin Endocr Metab.

[19] O'Dell SD, Miller GJ, Cooper JA, Hindmarsh PC, Pringle PJ (1997). Apal polymorphism in insulin-like growth factor II (IGF2) gene and weight in middle-aged males.. Int J Obes Relat Metab Disord.

[20] Deal C, Ma J, Wilkin F, Paquette J, Rozen F (2001). Novel promoter polymorphism in insulin-like growth factor-binding protein-3: correlation with serum levels and interaction with known regulators.. J Clin Endocr Metab.

[21] World Health Organization (1981). Histological typing of lung tumors (2nd ed.).

[22] Patel, Lim DS, Reddy D, Nagueh SF, Lutucuta S (2000). Variants of trophic factors and expression of cardiac hypertrophy in patients with hypertrophic cardiomyopathy.. J Mol Cell Cardiol.

[23] Ren Z, Cai Q, Shu XO, Cai H, Li C (2004). Genetic polymorphisms in the IGFBP3 gene: association with breast cancer risk and blood IGFBP-3 protein levels among Chinese women.. Cancer Epidemiol Biomarks Prev.

[24] Chen X, Guan J, Song Y, Chen P, Zheng H (2008). IGF-I (CA) repeat polymorphisms and risk of cancer: a meta-analysis.. J Hum Genet.

[25] Khan N, Afaq F, Saleem M, Ahmad N, Mukhtar H (2006). Targeting multiple signaling pathways by green tea polyphenol (−)-epigallocatechin-3-gallate.. Cancer Res.

[26] DeLellis K, Ingles S, Kolonel L, McKean-Cowdin R, Henderson B (2003). IGF1 genotype, mean plasma level and breast cancer risk in the Hawaii/Los Angeles multiethnic cohort.. Br J Cancer.

[27] Jernstrom H, Chu W, Vesprini D, Tao Y, Majeed N (2001). Genetic factors related to racial variation in plasma levels of insulin-like growth factor-1: implications for premenopausal breast cancer risk.. Mol Genet Metab.

[28] Gaunt TR, Cooper JA, Miller GJ, Day IN, O'Dell SD (2006). IGF2 polymorphisms are associated with hepatitis B virus clearance and hepatocellular carcinoma.. Biochem Biophys Res Commun.

[29] Gaunt TR, Cooper JA, Miller GJ, Day IN, O'Dell SD (2001). Positive associations between single nucleotide polymorphisms in the IGF2 gene region and body mass index in adult males.. Hum Mol Genet.

[30] Khan N, Afaq F, Saleem M, Ahmad N, Mukhtar H (2003). Insulin-like growth factor-binding protein-3 gene -202 A/C polymorphism is correlated with advanced disease status in prostate cancer.. Cancer Res.

[31] Schildkraut JM, Demark-Wahnefried W, Wenham RM, Grubber J, Jeffreys AS (2005). IGF1 (CA)19 repeat and IGFBP3 -202 A/C genotypes and the risk of prostate cancer in Black and White men.. Cancer Epidemiol Biomarkers Prev.

[32] Jian L, Binns CW, Lee AH (2006). Validity of a food-frequency questionnaire for elderly men in southeast China.. Public Health Nutr.

[33] Ogawa K, Tsubono Y, Nishino Y, Watanabe Y, Ohkubo T (2002). Validation of a food-frequency questionnaire for cohort studies in rural Japan.. Public Health Nutr.

[34] Hodgson JM, Chan SY, Puddey IB, Devine A, Wattanapenpaiboon N (2004). Phenolic acid metabolites as biomarkers for tea- and coffee-derived polyphenol exposure in human subjects.. Br J Nutr.

[35] Chen KY, Chang CH, Yu CJ, Kuo SH, Yang PC (2005). Distribution according to histologic type and outcome by gender and age group in Taiwanese patients with lung carcinoma.. Cancer.

[36] Chang CH, Hsiao CF, Chang GC, Tsai YH, Chen YM (2009). Interactive effect of cigarette smoking with human 8-oxoguanine DNA N-glycosylase 1 (hOGG1) polymorphisms on the risk of lung cancer: a case-control study in Taiwan.. Am J Epidemiol.

